# In situ architecture of a nucleoid-associated biomolecular co-condensate that regulates bacterial cell division

**DOI:** 10.1073/pnas.2419610121

**Published:** 2024-12-31

**Authors:** Peng Xu, Dominik Schumacher, Chuan Liu, Andrea Harms, Marcel Dickmanns, Florian Beck, Jürgen M. Plitzko, Wolfgang Baumeister, Lotte Søgaard-Andersen

**Affiliations:** ^a^Department of Molecular Structural Biology, Max Planck Institute of Biochemistry, Martinsried 82152, Germany; ^b^Department of Ecophysiology, Max Planck Institute for Terrestrial Microbiology, Marburg 35043, Germany; ^c^Research Group CryoEM Technology, Max Planck Institute of Biochemistry, Martinsried 82152, Germany

**Keywords:** liquid-liquid phase separation, biomolecular condensate, bacterial cell division, correlative cryo-electron tomography, cryo-electron microscopy

## Abstract

In most bacteria, cell division depends on the tubulin-homolog FtsZ that polymerizes in a GTP-dependent manner to form the cytokinetic Z-ring at the future division site. Subsequently, the Z-ring recruits, directly or indirectly, all other proteins of the divisome complex that executes cytokinesis. A critical step in this process is the precise positioning of the Z-ring at the future division site. While the divisome proteins are generally conserved, the regulatory systems that position the Z-ring are more diverse. However, these systems have in common that they modulate FtsZ polymerization. In *Myxococcus,* PomX, PomY, and PomZ form precisely one MDa-sized, nonstoichiometric, nucleoid-associated assembly that spatiotemporally guides Z-ring formation. Here, using cryo-correlative light and electron microscopy together with in situ cryoelectron tomography, we determine the PomXYZ assembly’s architecture at close-to-live conditions. PomX forms a porous meshwork of randomly intertwined filaments. Templated by this meshwork, the phase-separating PomY protein forms a biomolecular condensate that compacts and bends the PomX filaments, resulting in the formation of a selective PomXYZ co-condensate that is associated to the nucleoid by PomZ. These studies reveal a hitherto undescribed supramolecular structure and provide a framework for understanding how a nonstoichiometric co-condensate forms, maintains number control, and nucleates GTP-dependent FtsZ polymerization to precisely regulate cell division.

## Significance

Biomolecular condensates are essential for spatially organizing cellular reactions in bacteria and eukaryotic cells. Nevertheless, their mechanism remains poorly understood. Using cryoelectron tomography, we focused on the PomXYZ system that positions the cytokinetic FtsZ-ring in *Myxococcus*. We demonstrate that PomX forms a single copy per cell of a highly variable, porous meshwork of randomly intertwined filaments akin to a tumbleweed-like architecture. The PomY biomolecular condensate is templated by and embedded within the PomX meshwork, resulting in the formation of a selective PomXY co-condensate that is associated with the nucleoid by PomZ. These findings reveal a hitherto undescribed supramolecular structure and provide a framework for understanding how a nonstoichiometric co-condensate forms, maintains tight number control, and nucleates FtsZ polymerization.

Cell division is foundational to proliferation in all organisms. In most bacteria, cell division depends on the tubulin-homolog FtsZ (1, 2). At the division site, FtsZ, in a GTP-dependent manner, polymerizes to form the cytokinetic Z-ring (1). The Z-ring is responsible for recruiting, directly or indirectly, all other proteins of the divisome complex that executes cytokinesis (1). A critical step in this process is the precise positioning of the Z-ring at the future division site. Interestingly, while the divisome proteins are largely conserved (1), the regulatory systems that position the Z-ring are diverse and also incompletely understood (1, 3). However, they have in common that they modulate FtsZ polymerization (1, 3). Here, we focus on the PomXYZ cell division regulatory system in *Myxococcus xanthus*.

The PomX, PomY, and PomZ proteins form one MDa-sized nonstoichiometric nucleoid-associated cytoplasmic assembly per cell that spatiotemporally guides Z-ring formation at the nascent division site (4). PomX, PomY, and PomZ are present in multiple copies in this assembly and have distinct functions. PomX self-assembles to form a single complex per cell (4, 5). PomY interacts with and is enriched on this complex through multivalent interactions (4, 5). Because the local PomY concentration is above the critical saturation concentration (C*_sat_*) for phase separation, multivalent homotypic interactions allow PomY condensate formation via surface-assisted condensation on PomX (5), as has been described for phase-separating proteins that bind to DNA, microtubules, or the membrane (6–9). Consequently, PomY makes precisely one condensate per cell spatially templated by the single PomX complex. The ParA/MinD-like ATPase PomZ binds to the nucleoid and associates the PomXY assembly with the nucleoid (4, 10, 11). Moreover, by stimulating PomZ’s ATPase activity, PomX and PomY drive the translocation of the PomXYZ assembly across the nucleoid (4, 10, 11). While PomX is stably incorporated into this assembly, PomY and PomZ exchange with their cytoplasmic counterparts (5, 10). The translocation process eventually results in the localization of the PomXYZ assembly at midcell (4, 12), where the PomY condensate enriches FtsZ, nucleates its GTP-dependent polymerization, thereby stimulating Z-ring formation and cell division (4, 5, 11). During cytokinesis, the PomX complex undergoes a remarkable fission event, with each daughter cell acquiring a smaller PomX complex, while the PomY condensate disintegrates (5). Over the following cell cycle, the PomX complex accretes PomX molecules, allowing its size to double, and the PomY condensate reforms de novo on the PomX complex (5). Thus, the PomXYZ assembly is nonstoichiometric.

As in eukaryotic cells (13–16), biomolecular condensates have emerged as an important intracellular organizing modality in bacteria for processes including not only cell division but also chromosome segregation, transcription, RNA metabolism, cell polarization, CO_2_ fixation, and metabolism (5, 17–30). Biomolecular condensates are highly selective and spatially organize and compartmentalize cellular reactions by limiting the entry to specific molecules (13–16). In bulk solution, condensates form concentration-dependent above the C*_sat_*(13–16). As described for PomY (5), biomolecular condensates can also form through surface-assisted condensation, whereby binding of a phase-separating protein to a surface results in its local enrichment above C*_sat_*, thereby enabling condensate formation specifically on the surface (5–9, 31, 32). Above C*_sat_*, specific transient homotypic and/or heterotypic interactions between multivalent proteins result in the formation of dynamic, nonstoichiometric networks of interacting proteins that organize to form the spherical-to-spheroidal condensates (13–16).

Despite their importance in spatially organizing cellular reactions, only a few high-resolution biomolecular condensates have been visualized in situ using cryoelectron tomography (cryo-ET) (33–36). Thus, their architecture, biogenesis, number control, and mechanism of action remain poorly understood. Here, we leveraged state-of-the-art cryo-correlative light and electron microscopy (CLEM) with in situ cryo-ET to address these questions by focusing on the PomXYZ assembly at close-to-live conditions. This study uniquely elucidates the in situ architecture of a well-defined multicomponent biomolecular co-condensate, marking a significant advancement in the understanding of how biomolecular condensates organize cellular processes in space and time.

## Results

### The PomXYZ Assembly Adopts a Tumbleweed-Like Architecture In Situ.

**Fig. 1.**
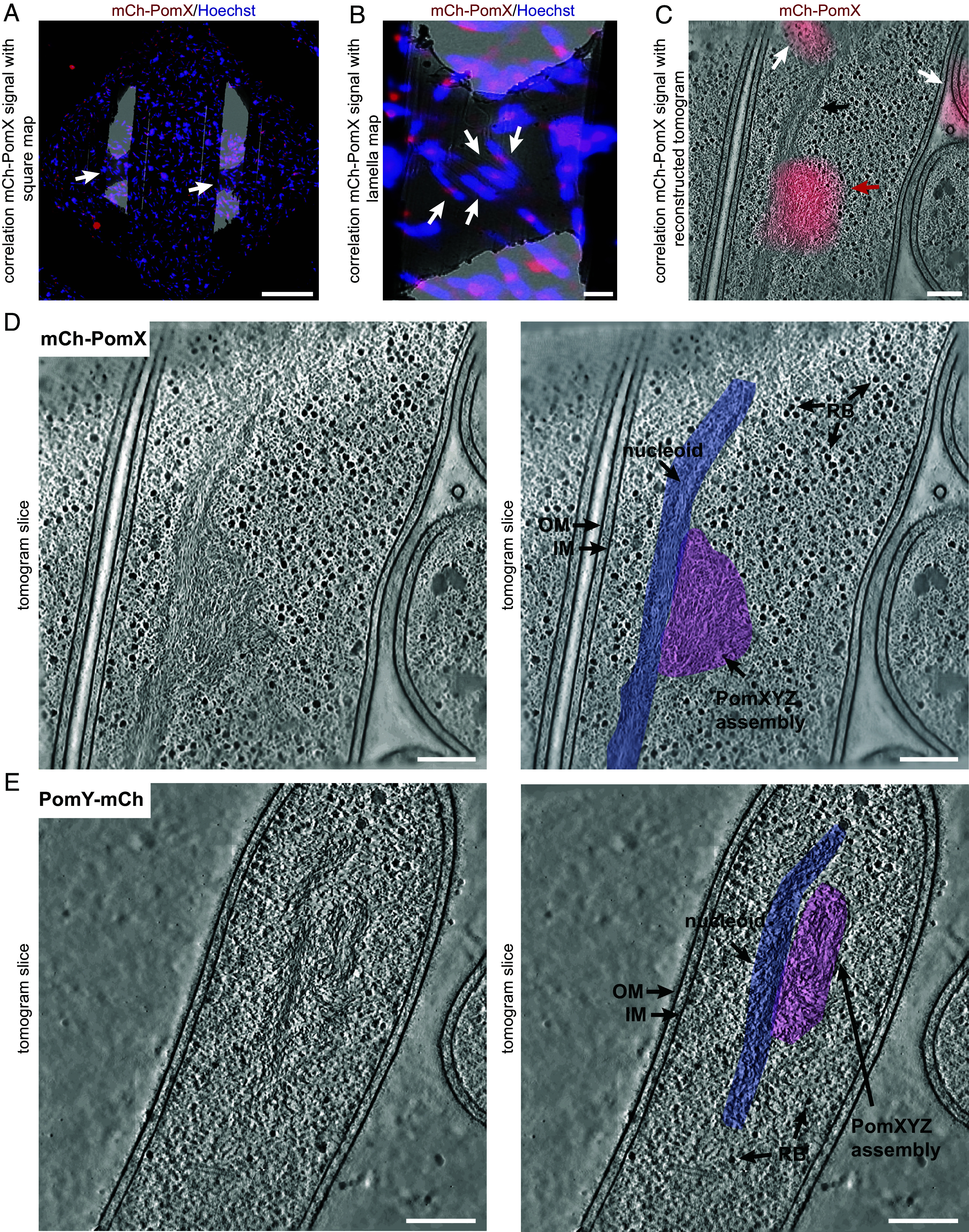
Identification of the PomXYZ assembly by CLEM and cryo-ET. (*A*) Correlation of mCh-PomX signal and Hoechst signal with cryo-ET square overview map at low magnification. White arrows indicate lamellae. (Scale bar, 20 µm.) (*B*) Correlation of mCh-PomX signal and Hoechst signal with cryo-ET lamella map at higher magnification. White arrows indicate target cells on lamella. (Scale bar, 2 μm.) (*C*) Correlation of mCh-PomX signal with a reconstructed tomographic slice. The black arrow indicates elongated filament bundles, white arrows mCh-PomX signals from outside the focal plane of cells removed by FIB milling, and red arrow mCh-PomX target signal. (Scale bar 100 nm.) (*D* and *E*). *Left*: Single image slice of a denoised tomogram of cells expressing mCh-PomX (*D*) or PomY-mCh (*E*). (Scale bar, 100 nm.) *Right*: The same images as on the *Left*; the nucleoid area is indicated in light blue and the PomXYZ assembly area in light purple, outer membrane (OM), inner membrane (IM), and ribosome (RB).

In vitro, purified PomX self-assembles into filaments, which by negative-stain transmission EM have a width of 8 to 10 nm and a length of several µm (4, 5, 10), while PomY alone under crowding conditions or at high concentrations forms µm-sized electron-dense, amorphous, spherical condensates (5); in the presence of the PomX filaments, the PomY condensates wet and bundle these filaments (5). These in vitro structures, however, do not match the sizes of the spherical-to-spheroidal PomX and PomY clusters observed in vivo by high-resolution structured illumination microscopy (SIM) (5). Therefore, to investigate the architecture of the PomXYZ assembly at close-to-live conditions, we used CLEM combined with cryo-ET on vitrified *M. xanthus* cells using two strains expressing either an active mCherry (mCh)-PomX or an active PomY-mCh fusion at native levels as the only source of PomX or PomY(5). Before vitrification, the nucleoid was stained with Hoechst for more precise CLEM. Due to the thickness of the sample, vitrified cells were thinned by cryo-focused ion beam (FIB) milling to generate 80 to 120 nm-thick lamellae before cryo-ET data acquisition (Fig. 1 *A* and *B* and *SI Appendix*, Fig. S1*A*). Using this workflow, we identified a single spherical-to-spheroidal area of intertwined filaments per cell that perfectly correlated with the mCh-PomX signal (n = 33) (Fig. 1 *C* and *D*) and the PomY-mCh signal (n = 17) (Fig. 1*E*) in the tomograms. In the tomograms (n = 50), this structure was immediately adjacent to a structure composed of longitudinally aligned filaments running along the long cell axis and which correlated with neither the mCh-PomX nor the PomY-mCh signal (Fig. 1 *C*–*E*). Because the PomXYZ assembly associates with the nucleoid, we speculated that this structure was the nucleoid. To test this idea, we repeated the CLEM/FIB milling/cryo-ET workflow on Hoechst-stained Δ*pomX* cells, which lack entirely the PomXYZ assembly (4). In the tomograms of Δ*pomX* cells (n = 27), the elongated filamentous structure remained and correlated perfectly with the Hoechst signal; by contrast, the structure containing the intertwined filaments was absent (*SI Appendix*, Fig. S1 *B* and *C*). Finally, in agreement with previous fluorescence microscopy-based results in which moderately overexpressed mCh-PomX formed elongated clusters (4, 5), an elongated meshwork of intertwined filaments was clearly visible in the tomograms (n = 28) when mCh-PomX was moderately overexpressed, while the elongated filamentous structure remained unaltered (*SI Appendix*, Fig. S1*D*). We conclude that the structure containing the intertwined filaments is the PomXYZ assembly, PomX as well as PomY are present within this structure, its filamentous part are PomX filaments, and the assembly is in close contact with the nucleoid, which is visible as an elongated structure of aligned filaments.

**Fig. 2.**
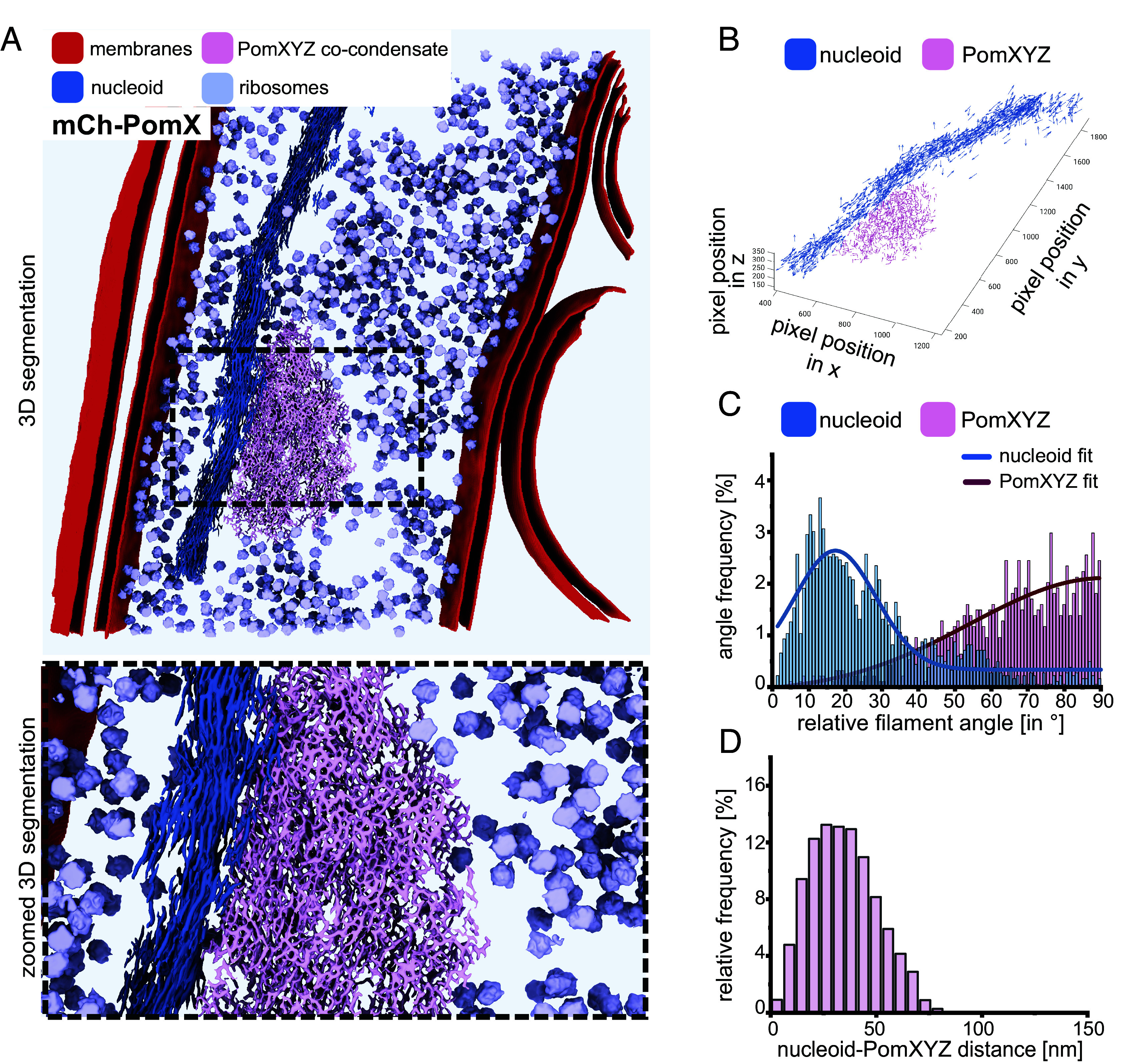
The PomXYZ co-condensate is selective and excludes ribosomes. (*A*) 3D rendering of features from the tomogram of Fig. 1*D* (*Upper* part) expressing mCh-PomX. *Lower* part, zoomed-in view of a boxed region from the *Upper* part. (*B*) 3D plot of individual nucleoid filament fragments and PomX filament fragments in the PomXYZ co-condensate. Arrows indicate orientation of individual filaments. (*C*) Histogram of the angles between individual nucleoid and PomX filament fragments to their corresponding averaged orientation. (*D*) Distribution of distances between nucleoid and PomXYZ co-condensate.

Tomograms of the PomXYZ assembly demonstrated that it has a remarkable three-dimensional structure akin to a tumbleweed, i.e. it is spherical-to-spheroidal, porous, and contains a filamentous meshwork of seemingly randomly intertwined filaments, which our data suggest are PomX filaments (Figs. 1 *D* and *E* and 2*A*). In agreement with SIM of the mCh-PomX cluster in vivo (5), the PomXYZ assembly in the tomograms spans several 100 nm along the long and short axes and the dimensions of the assembly vary between cells, as does the filament orientation (Fig. 1 *D* and *E*). Thus, the PomXYZ assembly does not have a precise three-dimensional architecture. In vitro, PomX self-assembles into long filaments; however, the tilt series’ resolution is insufficient to resolve whether the three-dimensional meshwork of PomX filaments is composed of long filaments that bend and curve in three dimensions or of short, seemingly randomly connected filaments.

### The PomXYZ Assembly Is a Selective Co-Condensate.

Next, we analyzed the properties of the PomXYZ assembly in more details using Convolutional Neural Network (CNN) training to trace filament structures (*SI Appendix*, Fig. S2*A*) (*SI Appendix*, *Supplementary Materials and Methods*). Briefly, we initially identified regions in a subset of the tomograms that contained the PomXYZ assembly and/or the nucleoid. Subsequently, we extracted images that contained the filaments of interest. Each PomX and nucleoid filament in these images was manually annotated to create a robust training set for the neural network, ultimately enabling the trained CNN to automatically trace and segment PomX filaments throughout an entire Pom assembly as well as nucleoid filaments throughout an entire nucleoid with high precision across tomograms.

We identified ribosome coordinates within tomograms through template matching, culminating in an overall 3D model of tomogram regions (Fig. 2*A* and Movie S1). Subtomogram averaging refined the details of ribosomes in situ to a resolution of 6Å, where features of rRNA and proteins were well resolved, revealing the unambiguous positioning of ribosomal protein subunits and rRNA (*SI Appendix*, Fig. S3 *A* and *B* and Table S1). This confident result enabled us to perform further spatial analyses in which we determined a mean nearest center-to-center distance between ribosomes of 26.2 nm (*SI Appendix*, Fig. S2*B*), consistent with findings in *Escherichia coli* (21.6 nm) and human cells (25.4 nm) (37, 38). Calculation of the distance between ribosomes and the PomXYZ assembly demonstrated that less than 1% of ribosomes were found in close proximity to a PomXYZ assembly (minimum distance=0 nm), while the peak distance was 176 nm (*SI Appendix*, Fig. S2 *C* and *D*). Importantly, no ribosomes were observed within the assemblies (Fig. 2*A* and *SI Appendix*, Fig. S2*D*). These two observations suggest that ribosomes, rather than directly interacting with the assemblies, are excluded from the PomXYZ assemblies. These observations also strongly suggest that the PomXYZ assembly function as a selective barrier. Based on these findings, we surmise that the PomXYZ assembly is a multicomponent co-condensate with selectivity and only allows the enrichment of specific molecules, a hallmark of biomolecular condensates. The mean nearest distance between the PomXYZ co-condensate and the cytoplasmic membrane was ~150 nm, and no PomXYZ co-condensates were identified that interacted directly with the cytoplasmic membrane (*SI Appendix*, Fig. S2*E*).

Using the CNN traced filaments, we analyzed the filament organization of the nucleoid and the PomXYZ co-condensate by fragmenting the filaments into equal-length vectors and examined their orientation. Subsequently, we calculated an average vector representing the average filament orientation and then the angle between each individual vector and this average. A distribution dominated by small angles indicates a higher degree of alignment and, thus straighter filaments, while a distribution dominated by large angles reflects filaments that are more randomly oriented and bent. Through this analysis, we found a high degree of orientational ordering of nucleoid filaments, with filament fragments having a narrow angle distribution, peaking at 17.3°, indicative of a highly organized structure in which the filaments are aligned (Fig. 2 *B* and *C*). By contrast, the angle distribution of filament fragments within the PomXYZ co-condensate was significantly (*P* < 0.0001) broader compared to that of the nucleoid, indicating a more disordered and bent filament arrangement with little orientational ordering (Fig. 2 *B* and *C*). In agreement with the close contact between the PomXYZ co-condensate and the nucleoid, the distance of coordinates within the 3D-segmented PomXYZ co-condensate to the nucleoid varied from 0 to 101 nm and with a mean of 35 nm (Fig. 2*D*).

### The Phase-Separating PomY Protein Provides Compactness and Selectivity to the PomXYZ Co-Condensate.

**Fig. 3.**
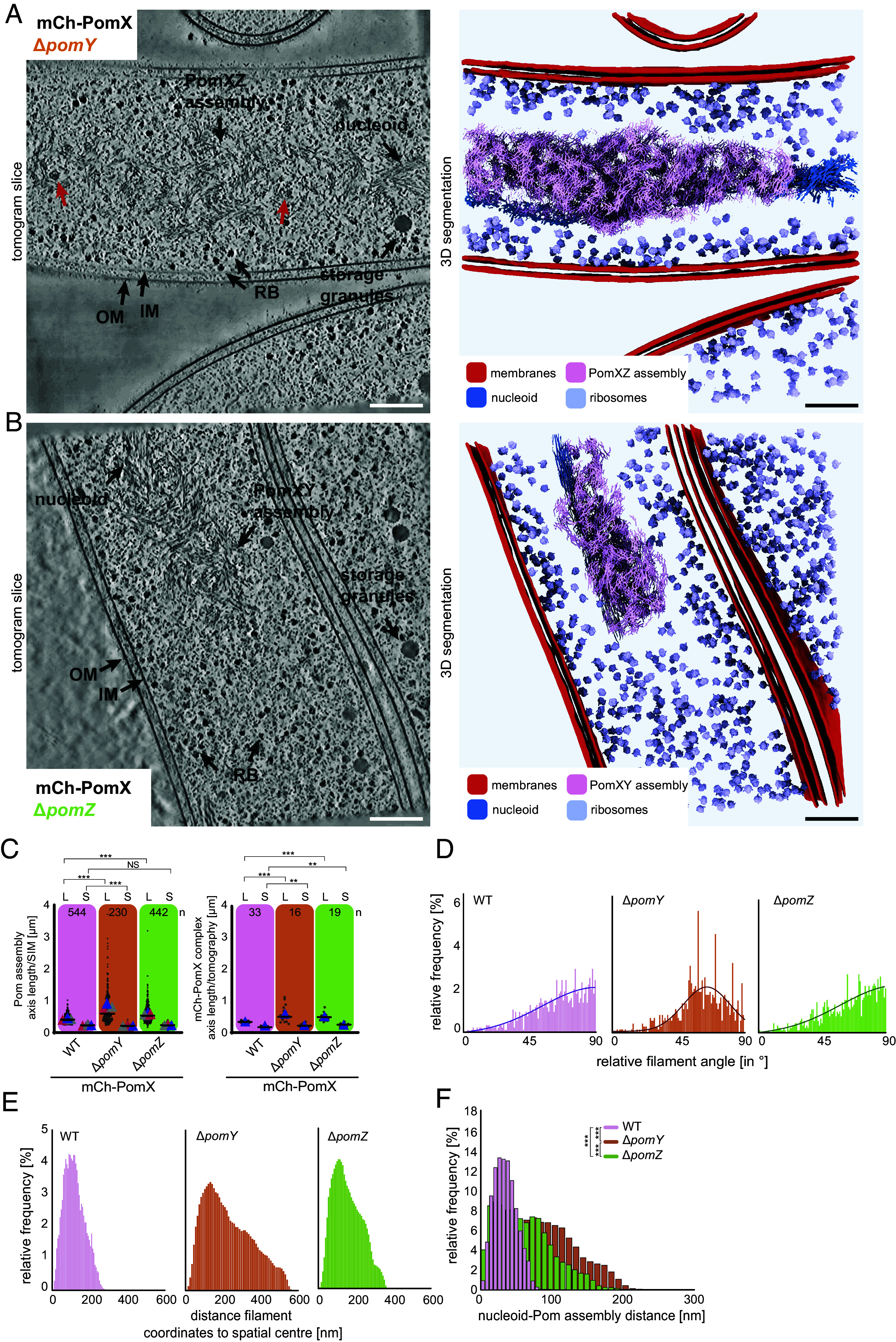
Function of PomY and PomZ in the PomXYZ co-condensate. (*A* and *B*) Tomographic slice of a Δ*pomY* (*A*) and Δ*pomZ* (*C*) cell (*Left*) and relative 3D segmentations of the tomograms (*Right*). Abbreviations and black arrows as in Fig. 1*D*. Red arrows point to larger protein densities inside the PomXZ assembly. (Scale bar, 100 nm.) (*C*) Quantification of the long (L) and short (S) axes of mCh-PomX complexes based on SIM images (*Left*) and Pom assemblies based in tomograms (*Right*). Number of cells and tomograms analyzed (n) is indicated. Colored triangles indicate the mean from three biological replicates and black line the median. Not significant (NS) *P* > 0.05, ** *P* ≤ 0.01, *** *P* ≤ 0.0001 based on ANOVA one-way test. (*D*) Histogram of the relative angle between individual Pom filament fragment orientation against the average filament orientation in strains of the indicated genotypes. (*E*) Histogram of the distances between Pom coordinates to its assembly spatial center. (*F*). Distribution of distances between nucleoid and Pom assembly in cells of indicated genotypes. *** *P* ≤ 0.0001 based on ANOVA one-way test.

In the absence of PomY or PomZ, the respective PomXZ or PomXY assemblies still form (4). To elucidate the structural and functional contributions of PomY and PomZ to the PomXYZ co-condensate, we determined the architecture of Pom assemblies lacking either PomY or PomZ in strains expressing mCh-PomX at native levels. By SIM and cryo-ET, the PomXZ assemblies were significantly larger than the PomXYZ co-condensates (Fig. 3 *A* and *C*). Consistently, the 3D reconstructions of PomXZ assemblies revealed that they were significantly elongated and less spherical compared to the PomXYZ co-condensates (Figs. 2*A* and 3 *A* and *C* and Movie S2). Intriguingly, the PomXZ assemblies contained large protein densities (Fig. 3*A*) and even ribosomes (*SI Appendix*, Fig. S2*F*), suggesting that these assemblies are less selective than the PomXYZ co-condensates. Also, while we observed that the filaments within the PomXYZ co-condensate were distinctly bent (Fig. 1 *D* and *E*), sometimes even forming a vortex-like pattern (*SI Appendix*, Fig. S4*A*), the filaments in the PomXZ assembly appeared straighter (Fig. 3*A* and *SI Appendix*, Fig. S4*B*). To quantitatively assess these differences, we applied the CNN-based method described above to trace the filaments and then analyzed the angle distribution of filament fragments within the PomXZ assembly. The angle distributions in the PomXZ and PomXYZ assemblies were significantly different (*P* < 0.001, peaking at 58° and 90° relative to the average orientation, respectively (Fig. 3*D*). These results indicate that, in the absence of PomY, the PomX filaments are more aligned, straighter, and less bent. Tracking the distance of filament coordinates to the spatial center of an assembly, we measured a peak distance of 151/105 nm and a maximum distance of 567/297 nm for PomXZ/PomXYZ (Fig. 3*E*). Thus, the density of PomX fragments is higher in the PomXYZ co-condensate compared to the PomXZ assembly. Finally, the mean distance of coordinates within 3D-segmented PomXZ assemblies to the nucleoid was increased to 80 nm (35 nm for PomXYZ co-condensate), and varied from 0 to 240 nm (Fig. 3*F*). The nucleoid width was unchanged in the absence of PomY (*SI Appendix*, Fig. S2*G*) suggesting that the increased distance of the PomXZ assembly to the nucleoid is a consequence of alterations of the Pom assembly architecture. We conclude that PomY i) contributes significantly to the architecture of the PomXYZ co-condensate by compacting and bending the PomX filaments, ii) provides the PomXYZ co-condensate with selectivity, and iii) assists in associating the co-condensate to the nucleoid. Based on sequence analysis and structural modeling, PomY does not contain a DNA binding domain. We speculate that the abnormal shape and volumetric expansion of the PomXZ assembly could cause the increased distance to the nucleoid.

By SIM and cryo-ET, the PomXY assemblies were slightly larger than the PomXYZ co-condensates but smaller than the PomXZ assemblies (Fig. 3 *B* and *C*). Consistently, tomograms of PomXY assemblies revealed that they were elongated and less spherical compared to the PomXYZ co-condensates, while PomX filament spacing appeared unaltered (Fig. 3*B* and Movie S3). Notably, the PomXY assemblies retained their selectivity as ribosomes and other large protein densities were not observed in their interior (Fig. 3*B* and Movie S3). Qualitatively, the PomX filaments in the PomXY assemblies appeared similarly bent as the filaments in the PomXYZ co-condensate and sometimes also formed a vortex-like pattern (Fig. 3*B* and *SI Appendix*, Fig. S4*C*). In agreement with these observations, the angle distribution of filament fragments within the PomXZ and PomXYZ assemblies were not significantly different (*P* = 0.7432) (Fig. 3*D*). Thus, PomZ does not contribute to the bending of the PomX filaments within the PomXYZ co-condensate. The density of PomX fragments in the PomXY assemblies was slightly lower than in the PomXYZ co-condensates with a peak distance of 107/105 nm and a maximum distance of 364/297 nm for PomXY/PomXYZ to their spatial centers (Fig. 3*E*). Finally, the mean distance of coordinates within 3D-segmented PomXY assemblies to the nucleoid was increased to 61 nm (35 nm for PomXYZ co-condensates) and varied from 0 to 199 nm (Fig. 3*F*). As in the case of cells lacking PomY, the nucleoid width was unchanged in the absence of PomZ (*SI Appendix*, Fig. S2*G*). We conclude that PomZ i) contributes to maintaining PomXYZ co-condensate size, and ii) assists in maintaining a close association of this co-condensate with the nucleoid. The latter conclusion agrees with PomZ being a DNA-binding protein and its DNA-dependent ATP hydrolysis enabling PomXYZ co-condensate translocation across the nucleoid (4, 10).

The width of the PomX filaments (~5 nm) was similar in all three assemblies (*SI Appendix*, Fig. S2*H*). Despite PomY and PomZ being part of the tumbleweed-like PomXYZ co-condensate and interacting with PomX, densities corresponding to these two proteins were not distinguished in the tomograms, likely because of their small sizes (PomY, 682 residues; PomZ, 319 residues) and the densely packed in situ environment.

### A Repeating Tetrameric Unit Forms the PomX Filaments.

Next, we attempted to structurally characterize the PomX filament. However, subtomogram averaging of the in situ PomX filaments was not possible due to their highly bent conformation and the dense cellular environment. Therefore, we purified full-length PomX-His_6_ and performed cryoelectron microscopy single particle analysis (cryo-EM SPA) on the self-assembled filaments (*SI Appendix*, Fig. S5 *A* and *B* and Table S2). Due to the flexibility of the filaments, we were only able to determine the PomX filament structure at a final resolution of 10Å. The density map suggests that the PomX filament comprises two parallel arrays with a total width of ~5 nm and several electron densities connecting the two arrays (*SI Appendix*, Fig. S5*B*). Thus, the PomX filaments have a similar width in vivo and in vitro.

**Fig. 4.**
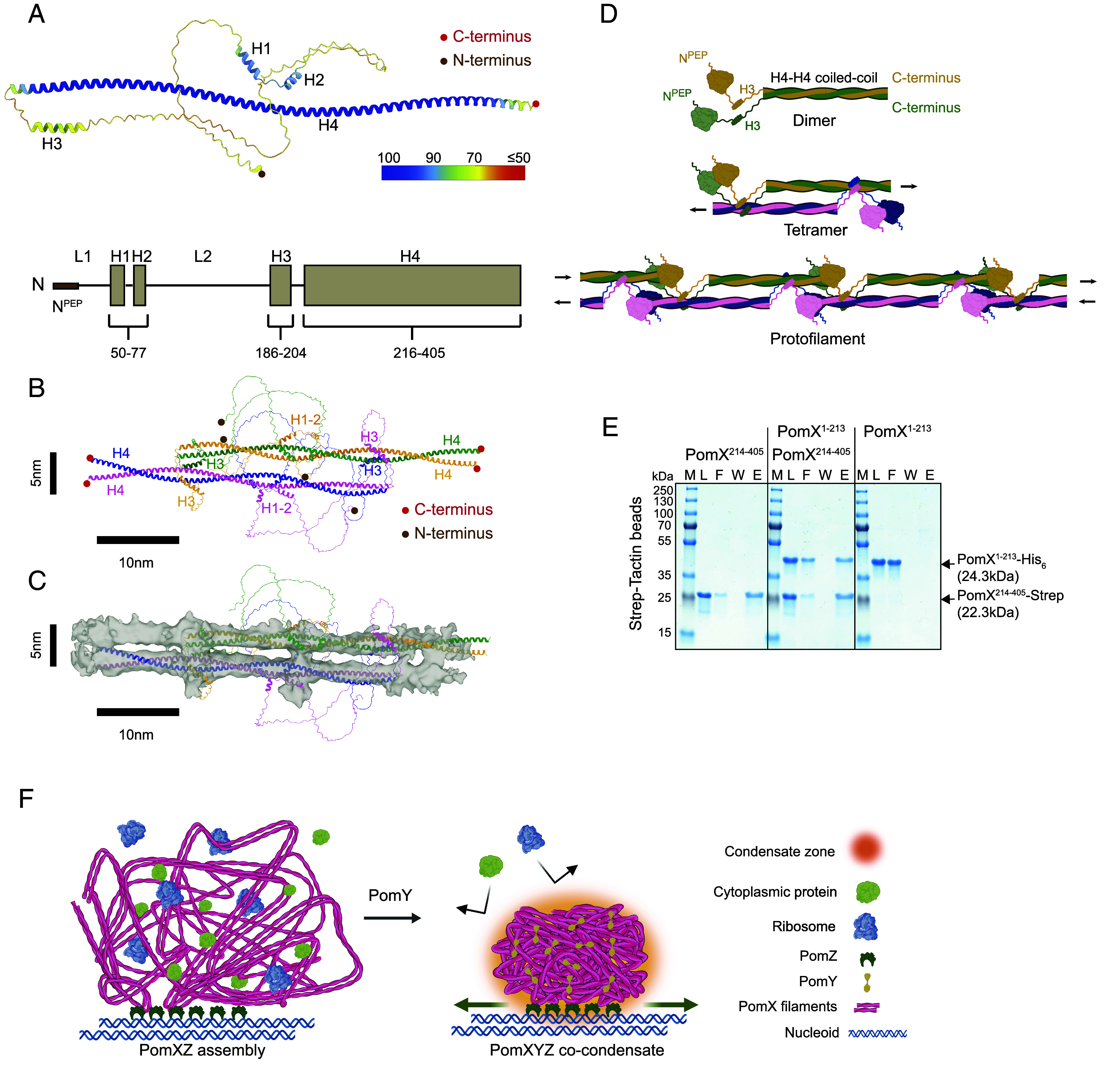
A repeating tetrameric unit forms the PomX filaments. (*A*) *Upper* part: AlphaFold-Multimer prediction of PomX monomer structure; model is colored by the predicted Local Distance Difference Test (pLDDT) score. Scores are indicated by blue to red color gradient. The N and C termini are indicated. *Lower* part: Schematic of the sequence and structure features of PomX based on AlphaFold-Multimer prediction. (*B*) Proposed structural model of PomX tetramer based on AlphaFold-Multimer prediction, colored by chain. The N and C termini are indicated. (*C*) Docking of proposed structural model of PomX tetramer into the cryo-EM SPA density map of PomX filament. Colors as in *B*. (*D*) Model of PomX self-assembly and elongation. Black arrows indicate filament’s directions. (*E*) In vitro pull-down experiments with purified PomX^214-405^-Strep and PomX^1-213^-His_6_. Instant Blue-stained SDS-PAGE shows load (L), flow-through (F), wash (W), and elution (E) fractions using Strep-Tactin beads in pull-down experiments with 10 µM of the indicated proteins alone or premixed as indicated on top. Molecular size markers are shown in lanes marked M. Note that PomX^1-213^-His_6_ migrates aberrantly and according to a higher molecular weight (10). All samples were analyzed on the same gel and black lines are included for clarity. (*F*) Integrative hypothetical model of PomXYZ co-condensate. Green arrows indicate PomXYZ co-condensate translocation across the nucleoid.

Due to the relatively low resolution of the cryo-EM SPA density map, we were unable to obtain a high-resolution structure of the PomX filament. To overcome this limitation, we used AlphaFold-Multimer to predict potential configurations of PomX. Based on AlphaFold-Multimer, the PomX monomer comprises four α-helical regions (H1, H2, H3, H4), including the long C-terminal H4 predicted to engage in α-helical coiled-coil formation, as well as two unstructured regions, L1, which contains the N-terminal 22 residues (N^PEP^) that are required and sufficient for stimulation of PomZ ATPase activity by PomX (10), and L2 (Fig. 4*A*). In this structural model, H4 is predicted with high confidence (>90%) (Fig. 4*A*), while the confidence scores for H1, H2, H3, L1, and L2 are lower, indicating a more disordered N-terminal region (Fig. 4*A*).

Subsequently, we used AlphaFold-Multimer to model different oligomeric states of PomX, ranging from dimers to octamers (*SI Appendix*, Fig. S6 *A*–*D*). Remarkably, two PomX tetramer models had highly similar rigid bodies, mainly differed in the mostly unstructured N-terminal region (models a and b in *SI Appendix*, Fig. S6*D*), and fitted well into the single particle density map (Fig. 4 *B* and *C*). Focusing on model a, i) two PomX molecules interact via their H4 helices to form a 28-nm long H4–H4 α-helical coiled-coil; ii) two H4–H4 coiled-coil structures align, without interacting, antiparallel and staggered over a region of 19 nm giving rise to a unit with a width of ~5 nm, matching well the PomX filaments’ width in vitro based on cryo-EM SPA (Fig. 4*B*) and in vivo based on cryo-ET (*SI Appendix*, Fig. S2*H*); and, iii) two pairs of H3-helices connect the two H4–H4 coiled-coil structures (Fig. 4 *B* and *C* and *SI Appendix*, Fig. S5*C*). When this model was fitted into the single particle density map (Fig. 4*C*), the two antiparallel H4–H4 coiled-coil structures fitted well with the two parallel arrays, and the regions corresponding to the two pairs of connecting H3-helices aligned with two densities connecting the two arrays (Fig. 4*C*). The N-terminal, largely unstructured 185 residues seemingly lack interactions with the H4–H4 coiled-coil in the tetramer (Fig. 4 *B* and *C* and *SI Appendix*, Fig. S5 *C* and *D*).

Based on these observations, we propose that tetrameric PomX constitutes the repeat unit of the PomX filament and that the filament assembles by longitudinal, head-to-tail associations through interactions between the largely unstructured, N-terminal regions of one dimer and the C-terminal part of an H4–H4 coiled-coil of a flanking dimer (Fig. 4*D*). This model is supported by the observations that the truncated PomX variant containing residues 214-405 (PomX^214-405^) not only self-interacts in vitro (10) but also interacts with the PomX^1-213^ variant (Fig. 4*E*), while the PomX^1-213^ variant does not self-interact, and none of the two truncated variants self-assemble to form the PomX meshwork in vivo (10). Remarkably, the structural organization and assembly mechanism of PomX filaments echo those of intermediate filaments (39, 40). Intermediate filaments assemble from dimers formed by coiled-coil regions that align antiparallel to form tetramers. These tetramers then connect head-to-tail, facilitated by interactions between an unstructured N-terminal “head domain” and a coiled-coil C-terminal “tail domain”.

## Discussion

Traditionally, the function of proteins depends on their assembly into complexes with well-defined stoichiometry and structure. Here, we describe the architecture of the MDa-sized PomXYZ co-condensate, which has neither a well-defined stoichiometry nor a well-defined structure. Nevertheless, it executes the spatiotemporal regulation of cell division with exquisite precision.

We propose the following model for PomXYZ co-condensate architecture, biogenesis, number control, and mechanism of action (Fig. 4*F*). The PomX filament is built from a dimer-of-dimers tetrameric repeat unit (Fig. 4*D*). In vivo, the PomX assembles into a meshwork of intertwined, randomly oriented filaments with no overall spatial order akin to a tumbleweed-like architecture. Because cells contain precisely one copy of the PomX meshwork, we suggest nucleation of filament formation as the limiting factor for PomX self-assembly. Making filament nucleation the limiting factor compared to filament growth would help to ensure that only one PomX complex forms per cell. In agreement with the observations that PomX and PomY colocalize by fluorescence microscopy and the filamentous PomX meshwork templates PomY condensate formation in vivo (4, 5), the mCh-PomX and PomY-mCh signals both overlap with the tumbleweed-like structure. Thus, both proteins are present in this structure. These observations support that the surface-assisted phase separation by PomY occurs within the matrix of PomX filaments. The PomX filaments are significantly more compact and bent in the presence of PomY than in its absence. We suggest that PomY molecules connecting PomX filaments as well as the surface tension of the PomY condensate embedded within the filamentous PomX meshwork force compaction and bending of the PomX filaments, as described for synthetic condensates (41). This structural model also explains how the PomY condensate can accrete additional PomY molecules: As PomX filaments grow, more PomY binding sites become available, allowing PomY binding and integration into the PomY condensate. Importantly, the PomY condensate confers the PomXYZ co-condensate with selectivity. Finally, for the PomXYZ system to work reliably, PomY phase separation must be spatially restricted to ensure the formation of only one PomY condensate per cell. Our data support that precise control of the PomY condensate number per cell is guaranteed by the nucleation-limited formation of the single PomX meshwork, the surface-assisted condensation of PomY on this meshwork together with the low cellular PomY concentration (5). We previously suggested that the PomY condensate formed “on top of” a filamentous PomX complex. This model was based on the observation that PomY condensates in vitro wet the PomX filaments. The data presented here demonstrate that the PomY condensate is embedded within the meshwork of PomX filaments, emphasizing the importance of studying condensates at close-to-native conditions to obtain architectural and biogenesis insights. Finally, our data and previous findings suggest that the main functions of PomZ are to associate the PomXY assembly with the surface of the nucleoid and the ATP-powered translocation of the PomXYZ co-condensate across the nucleoid.

In vitro PomY condensates enrich FtsZ and nucleate its GTP-dependent polymerization, thereby enabling the formation of FtsZ filaments that extend beyond the condensates (5). Consistently, PomY is essential for cytokinesis to occur over the PomXYZ co-condensate and fluorescently labeled PomY and FtsZ colocalize at the division site (4). The structural underpinnings of these activities are not known. However, the architecture of the PomXYZ co-condensate and previous observations may provide a framework for beginning to understand how PomY within the PomXYZ co-condensate could stimulate FtsZ polymerization. Specifically, the PomXYZ co-condensate appears sufficiently porous to not only allow the entry and diffusion of FtsZ monomers within the co-condensate but also to accommodate nucleated FtsZ filaments. Furthermore, the porosity of the PomXYZ co-condensate could allow internal rearrangement of PomY molecules to enable FtsZ nucleated in the interior to polymerize and extend beyond the condensate. Interestingly, several biomolecular condensates in eukaryotic cells nucleate actin (8, 42–45) or tubulin (46–48) polymerization. The architecture of the PomXYZ co-condensate provides a structural framework for beginning to understand how these biomolecular condensates may function.

Our CLEM/cryo-ET workflow allowed the visualization of the in situ architecture of the PomXYZ co-condensate that was unexpected based on previous in vitro analyses, emphasizing the importance of investigating supramolecular assemblies’ structure–function relationship in their native context. The methodologies used in this study lay down a robust framework for analyzing the structural features of other biomolecular condensates.

## Materials and Methods

### *M. xanthus* Strains and Growth.

*M. xanthus* strains used in this study include SA4777 (Δ*mglA*; Δ*pomX*) (5), SA4797 (Δ*mglA*; Δ*pomX; attB::P_nat_mCh-pomX*) (4), SA7046 (Δ*mglA*; Δ*pomX;* Δ*pomY attB::P_nat_mCh-pomX*), SA7061 (Δ*mglA*; Δ*pomX;* Δ*pomZ attB::P_nat_mCh-pomX*) (10), SA7064 (Δ*mglA*; Δ*pomY; attB::P_nat_pomY-mCh*) (5), and SA9734 (Δ*mglA*; Δ*pomX; attB::P_pilA_mCh-pomX*) (5). All *M. xanthus* strains are nonmotile due to a deletion of *mglA* (49). *M. xanthus* was grown at 32 °C in 1% CTT broth (1% (w/v) Bacto Casitone, 10 mM Tris-HCl pH 8.0, 1mM K_2_HPO_4_/KH_2_PO_4_ pH 7.6, 8 mM MgSO_4_) or on 1% CTT 1.5% agar (50). Kanamycin, oxytetracycline, and gentamycin were added at concentrations of 50 µg/mL, 10 µg/mL, and 10 µg/mL, respectively. Growth was measured as an increase in optical density (OD) at 550 nm.

Protein sequences: PomX (MXAN_0636, Q1DEL9), PomY (MXAN_0634, Q1DEM1), and PomZ (MXAN_0635, Q1DEM0).

## Supplementary Material

Appendix 01 (PDF)

Movie S1. Sequential slices back and forth through a representative tomogram in cross-section view and the 3D rendering models for a cell expressing mCh-PomX.

Movie S2. Sequential slices back and forth through a representative tomogram in cross-section view and the 3D rendering models for a Δ*pomY* cell expressing mCh-PomX.

Movie S3. Sequential slices back and forth through a representative tomogram in cross-section view and the 3D rendering models for a Δ*pomZ* cell expressing mCh-PomX.

## Data Availability

The authors declare that all data supporting this study are available within the article, or in Supplementary Information provided with this article. Maps have been deposited in the EMDB under accession codes: in situ 70S ribosome (EMD-50504) (51) and PomX filaments (EMD-50515) (52). Maps used from previous studies were obtained from the EMDB-3493 (53). All materials used in the study are available from the corresponding authors. All other data are included in the manuscript and/or supporting information.
