# Supplementary material for: In situ architecture of a nucleoid-associated biomolecular co-condensate that regulates bacterial cell division
Source: Proc Natl Acad Sci U S A. 2024 Dec 31;122(1):e2419610121. doi: 10.1073/pnas.2419610121 (PMC11725790; doi:10.1073/pnas.2419610121)
Supplement: Supplementary file 1 — Appendix 01 (PDF) [file pnas.2419610121.sapp.pdf]

Supplementary Information for

In situ architecture of a nucleoid-associated biomolecular co-condensate that regulates bacterial cell division

Peng Xu[1, 4, 5], Dominik Schumacher[2, 4], Chuan Liu[1], Andrea Harms[2], Marcel Dickmanns[1], Florian Beck[1], Jürgen M. Plitzko[3], Wolfgang Baumeister[1,5] and Lotte Søgaard-Andersen[2, 5]

[1] Department of Molecular Structural Biology, Max Planck Institute of Biochemistry

Am Klopferspitz 18, 82152 Martinsried, Germany

[2] Department of Ecophysiology, Max Planck Institute for Terrestrial Microbiology

Karl-von-Frisch Str. 10, 35043 Marburg, Germany

[3] Research Group CryoEM Technology, Max Planck Institute of Biochemistry,

Am Klopferspitz 18, 82152 Martinsried, Germany

[4] these authors contributed equally

[5] Corresponding authors: E-mails, baumeist@biochem.mpg.de,sogaard@mpi-marburg.mpg.de, xupeng@biochem.mpg.de

This PDF file includes:

- Supplementary Materials and Methods
- Supplementary Figures 1 to 6
- Supplementary Tables 1 to 2
- Legends to Movies S1 to S3
- Supplementary References

Supplementary Materials & Methods

Structured illumination microscopy (SIM). SIM was performed as described (1). Briefly, exponentially growing cells were transferred to a coverslip mounted on a metallic microscopy slide and covered with a pre-warmed 1% agarose pad, supplemented with 0.2% CTT broth. Slides were covered with parafilm to retain humidity of the agarose, and live-cell imaging performed at 32°C. SIM was performed on a temperature-controlled AxioObserver with the Zeiss Elyra 7 Lattice SIM module with perfect focus and an α-Plan-Apochromat 63×/1.46 oil Korr M27. Images were recorded with a pco.edge 4.2 sCMOS camera (PCO). mCh-PomX images were acquired using a 561nm 500mW laser with 100ms exposure time and 1.5% laser power. DAPI images were acquired using a 405nm 50mW laser with 150ms exposure time and 5% laser power. SIM images were reconstructed from 13 frames without rescaling using Zen software (Zeiss). Image processing was performed with Metamorph v 7.5 (Molecular Devices). For image analysis, cellular outlines were obtained from phase-contrast images using Oufti (2) and manually corrected if necessary. Fluorescence microscopy image analysis was performed with a custom-made Matlab script (Matlab R2018a, MathWorks) as described (3).

In vitro pull-down experiments. $PomX^{1\text{-}213}$-His_6 and $PomX^{214\text{-}405}$-Strep were purified as described (3). For the pull-down experiments, proteins were dialyzed against buffer P (10mM HEPES/NaOH pH 7.0, 20mM NaCl, 0.02% Tween 20). 10µM protein alone or pre-mixed as indicated were incubated for 18hrs at 4°C and then for 1hr at 32˚C in buffer P in a total volume of 200µl and then applied to 30µl Strep-Tactin®XT beads (IBA Lifesciences) for 60min. Magnetic beads were washed five times with 200µl buffer P. Proteins were eluted with 100µl BXT buffer (100mM Tris-HCl pH 8.0, 150mM NaCl, 1mM EDTA, 50mM biotin) (IBA Lifesciences)). 10µl per sample were separated by SDS-PAGE and stained with Instant Blue (expedion).

Cryo-grid preparation. *M. xanthus* strains were grown in suspension culture as described. For plunge-freezing, cells were adjusted to an OD550 of 1.6. In order to stain DNA, 1× Hoechst 33258 was added for 30min prior to plunge-freezing. To reduce the formation of crystalline ice, glycerol was added to the media as cryo buffer with final concentration of 5% v/v. A total of 4 µL of the cell suspension were added to the carbon side of a glow-discharged Cu 200 mesh R2/1 Quantifoil grid and manually blot from the back of grid to remove excess liquid. Then 3µL cryo buffer was added to cells and immediately blotted by vitrobot mark IV with 90% humidity at 37°C, blot force and time were 10 and 10s. Cells were plunge-frozen in an ethane/propane mixture cooled to liquid nitrogen temperatures. Grids were clipped into homemade cutoff autogrid support ring and kept in liquid nitrogen to facilitate downstream handling.

Cryo-focused ion beam (FIB) milling and Cryo-fluorescence microscopy. Grids were mounted into an Aquilos cryo-focused ion beam/scanning electron microscope (FIB/SEM dual-beam microscope, Thermo Fisher Scientific). Liquid nitrogen temperature was kept during whole FIB operation. To improve sample conductivity and reduce curtaining artifacts during FIB milling, grids were first sputter-coated with platinum and then coated with organometallic platinum using the gas injection system. Mono-layer cells with several empty holes squares were chosen as targets for milling. Lamellae were prepared using Gallium ion beam at 30 kV and stage tilt angles of 13°. Rough milling was done with currents of 100pA until lamellae thickness around 800nm. After rough milling, grids were unloaded from FIB

and mounted into a Leica SP8 cryo-confocal microscope with a cryo-stage operated at liquid nitrogen temperature. The grid overview was first acquired using the brightfield path to locate individual lamella. Squares with lamellae were imaged with an XY-pixel size of 60 nm and total z-step size of 1.2μm under confocal path. Signals were acquired by exciting with 552nm laser for mCherry and 405nm laser for Hoechst 33258 together with brightfield. The raw stacks were deconvolved with HUYGENS software (Huygens Professional version 21.10, Scientific Volume Imaging) for improved signal precision.

Grids were loaded into FIB again for correlated fine milling. lamellae were imaged under SEM operated at 30kv, 50pA and stage tilt angles of 13°. About 10 specific features (empty holes, corner of lamella, et) were picked both from fluorescent stack and relative SEM image for correction with 3DCT software (v2.2.2). According to the correlative results, fine milling of the lamellae as gradually reduced to lower currents, done with 30pA for the final polishing step.

Cryo-electron tomography data collection. The cryo-ET tilt series were collected with a Titan Krios Microscope G2 (Thermo Fischer Scientific) operated at a voltage of 300 KV and equipped with a quantum postcolumn energy filter and a K2 Summit direct electron detector (Gatan Inc). 340× magnification square map images were acquired. Meanwhile, lamella maps were acquired under montage mode at 4800× magnification. Two rounds of correlation were performed to accurately locate interested areas. Firstly, picking the aforementioned specific features on square map to correlate with the respective fluorescent Z-projection images. By landmark correspondence function in FIJI, transformed fluorescence images to fit TEM square map. Secondly, correlated lamella map with fluorescent overlayed square map to obtain fluorescent overlayed lamella map. Hoechst stained DNA pattern should fit cells pattern on lamella precisely. After correlation, mCherry signal indicated PomXYZ co-condensate location.

Data collected with a dose-symmetric tilt scheme using SerialEM 3.8 (https://bio3d.colorado.edu/SerialEM/) after automated stigmation, coma-free alignment, and coma vs image shift compensation. The recording magnifications were set at 42,000 with a pixel size of 3.52Å, tilt range was + 53° to −67° with 3° steps starting at −7° to compensate for the pre-tilt. constant dose of 3.5e−/Å^2 for all tilts with a total dose of ~ 140e−/Å^2 and defocus at -5μm.

A small dataset contain 53 tilt series was collected by a Titan Krios G4 instrument at 300 kV equipped with a Selectris X energy filter and a Falcon 4i camera (Thermo Fisher Scientific). Tilt series were recorded using Tomo5 software package (Thermo Fisher Scientific). A dose-symmetric tilt scheme was used with an angular increment of 3°. Magnification was set as 42,000x with a pixel size of 2.93Å, a dose of 3.5e− /Å^2 per tilt and a target defocus range from −4.5 to −5μm. and total dose ~140e−/Å^2.

Tomogram reconstruction and segmentation. Data were processed using the TOMOMAN version 0.7 pipeline (https://github.com/williamnwan/TOMOMAN). After motion correction in MotionCor2 version 1.4.7 (4), bad tilts were removed after manual inspection using the TOMOMAN script. Tilt series were separated into odd and even tilts during motion correction for denoising, and the resulting stacks were processed using Cryo-CARE (5) and ISOnet (6). Tilt series were aligned with AreTomo version 1.3.3 (7). Tomogram reconstructions were

done with IMOD version 4.12.32 (8, 9) at bin4 for visualization and ribosome template matching, at bin2 for nucleoid and Pom assembly segmentation.

For the CNN-based segmentation of Pom assemblies, we first generated soft masks in Amira (Thermo Fisher Scientific) to exclude the membrane and background, isolating the nucleoid and Pom assembly areas for segmentation in EMAN2 (10). For the training of the CNN-based segmentation model in EMAN2, 400 regions, which did not contain nucleoid or Pom assembly structures, were selected as negative references to help the network identify areas without the target features. In parallel, 50 positive references were chosen to represent nucleoid regions, and 80 positive references were selected for Pom assembly areas. Each positive region was manually annotated to accurately represent the structural features of interest. These annotated regions were then used to train the neural network, enabling it to automatically trace and segment PomX and nucleoid filaments with high precision across the tomograms.

Ribosome locations were determined using the template matching routine from STOPGAP version 0.7 (11). A reference 70S prokaryotic ribosome (EMD-3493 (12)) was filtered to a 40 Å resolution to serve as the template. A cross-correlation threshold that resulted in most ribosome-like particles being included was determined through Chimera (13). Missing targets and false negative were manually modified in the particles list through TOM and AV3 toolboxes (14). Particles were extracted in Warp version 1.0.9 (15) and averaged in RELION 3.0 (16, 17). Membranes were detected using TomoSegMemTV (18) and manually optimized in Amira. Structure visualization and movie generation were conducted with UCSF ChimeraX (19).

Cryo-ET subtomogram averaging of ribosome. Tilt series collected from Titan Krios G4 with lamella thickness less than 130nm were applied to subtomogram averaging. Template matching was performed as described above. 14,212 particles were extracted from 24 tomograms by Warp(15) with a pixel size of 2.93Å and a box size of 198 pixels. 3D-classifications with a spherical mask 400 Å in radius was used in RELION 3.0 (16, 17) to align the subtomograms. 8,087 particles were retained and imported to M software (20) for geometric and CTF parameters refinement. After resolution convergence was reached, a final map of 6.0Å was obtained within the reconstruction mask with the 0.143 criterion.

Quantification and statistical analysis of cryo-ET data. Dimensions of the nucleoid and Pom assemblies were measured with IMOD (8, 9) measurement module. The distances between ribosomes and Pom assemblies were calculated primarily using the Matlab functions bwdist and ind2sub. This process started by plotting the ribosome map into a 3D volume after Relion alignment and shift correction. Using ind2sub to transfer ribosomes 3D volume to 3D index. The bwdist function was then employed to generate a distance matrix for segmentation of the Pom assemblies, then apply the distance matrix to ribosomes 3D index. The distances from the Pom assemblies to the nucleoid were calculated in a similar manner. The nearest centre-to-centre distances of ribosomes were calculated based on corrected coordinates post template matching and Relion alignment. The distribution of filament angles for the nucleoid and Pom assemblies was determined based on 3D segmentation from EMAN2, with the principal axis of fragments identified mainly through the Matlab regionprops3 function. The spatial center of the Pom assemblies was determined by

calculating the maximal and minimal values along the X, Y, and Z axes via 3D segmentation. Subsequently, the ind2sub function was used to convert the Pom assembly segmentation into coordinates to calculate distances to the centre point. All relevant MATLAB scripts for this process are made available on https://github.com/CryoXu/PomXYZ. P-values were calculated using the ANOVA One-Way Test (21).

Cryo-electron microscopy single particles analysis. PomX-His$_6$ was purified as described (22). 4µL of the PomX-His$_6$ solution was applied to glow-discharged, holey carbon-coated copper grids (R 1.2/1.3, 200 mesh, Quantifoil Micro Tools). The grids were then plunge-frozen in a liquid ethane/propane mixture with a Vitrobot Mark 4 (FEI, Thermo Fisher Scientific) under conditions of 4°C and 95% humidity. Blotting was conducted for 10 seconds at a blot force of 20. The front side of the grids was treated with Teflon, and the back side with filter paper. Subsequently, the grids were stored in liquid nitrogen. Image acquisition was performed using a Titan Krios microscope (FEI, Thermo Fisher Scientific) at 300kV, utilising a quantum postcolumn energy filter and a K2 direct electron detector (Gatan). Movies were acquired at a pixel size of 1.09Å at specimen level. A total dose of roughly 40 electrons was distributed over 40 frames. The nominal defocus range of the acquisition varied from 1 to 3.5µm.

Data processing for single particle analysis. Frame alignment was performed using Relion's Motioncor2 implementation with 10×10 patches in Relion4 (16). The micrographs were imported into CryoSPARC (23). The contrast transfer function (CTF) was determined using CryoSPARC Patch CTF. Micrographs with a defocus outside the range of 0 to 2µm and an estimated resolution over 5 Å were discarded using CryoSPARC Curate Exposures. Automated particle picking was performed with CryoSPARC Filament Tracer. This process extracted 1,991,224 particles with a box size of [400×400] pixels using CryoSPARC Extract Mics. The reference-free 2D classification in CryoSPARC, with the 'Align filament classes vertically' option on, was used to remove false positives and non-aligning particles, yielding 367,462 particles. Two rounds of 2D classification were performed, selecting the classes that clearly resembled two parallel lines (161,664 particles). From three initial model classes, the best-resolved class was chosen as the reference for refinement. The refinement of the consensus map was conducted with a soft binary mask around the structure. Next, NU-refinement (23) and flexible refinement (24) was performed. As the filament was elongated and flexible, the alignment was restricted to 10Å to prevent overfitting.

AlphaFold-Multimer modelling of PomX. Structures of PomX in various oligomeric states (monomer, dimer, trimer, tetramer, octamer) were predicted using AlphaFold-Multimer v2.3.1 (25, 26). The resulting 25 models of the tetramer and other oligomeric states were similar in confidence (pLDDT, ipTM) and consistently suggested the formation of H4-H4 α-helical coiled-coil dimers.

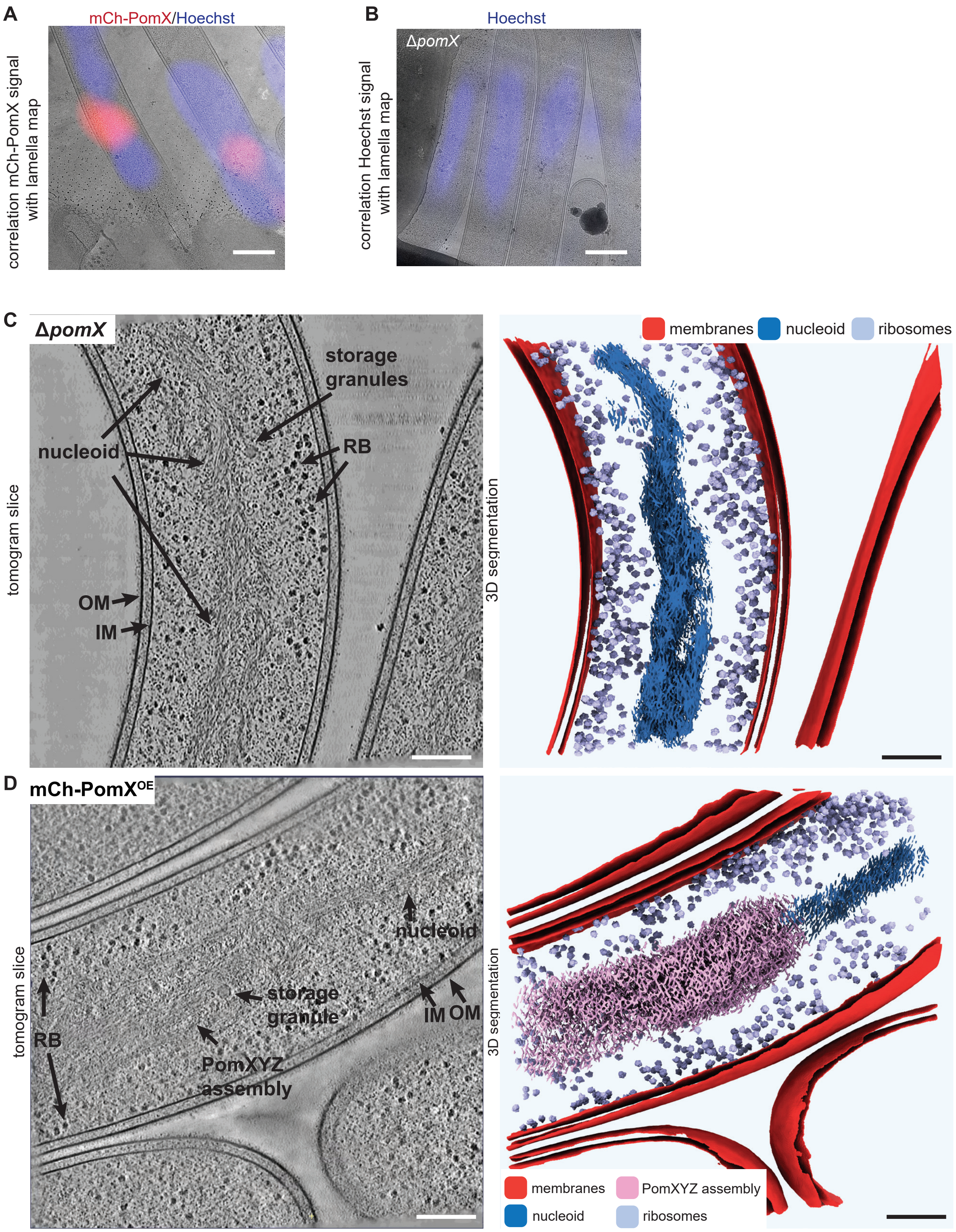
A
mCh-PomX/Hoechst
correlation mCh-PomX signal with lamella map
B
Hoechst
ΔpomX
correlation Hoechst signal with lamella map
C
ΔpomX
storage granules
nucleoid
RB
OM
IM
tomogram slice
membranes
nucleoid
ribosomes
3D segmentation
D
mCh-PomX^OE
nucleoid
storage granule
PomXYZ assembly
RB
IM
OM
tomogram slice
3D segmentation
membranes
PomXYZ assembly
nucleoid
ribosomes

Figure S1. The nucleoid is visible as an elongated structure of parallel filaments.
(A) Correlation of mCherry-PomX signal (red) and Hoechst signal (blue) with cryo-ET Zoomed-in lamella map. Scale bar 500nm.
(B) Correlation of Hoechst signal (blue) with cryo-ET lamella map of Δ*pomX* cells. Scale bar 500nm.
(C) Left: Slice of a denoised tomogram of a Δ*pomX* cell. Black arrows and abbreviations are as in Fig. 1D. Right: Relative 3D rendering of features from the tomogram. Scale bars 500nm.
(D) Left: Slice of a denoised tomogram of a cell moderately overexpressing mCh-PomX (PomX^{OE}). Black arrows and abbreviations are as in Fig. 1D. Right: Relative 3D rendering of features of the tomogram as on the left. Scale bars 100nm.

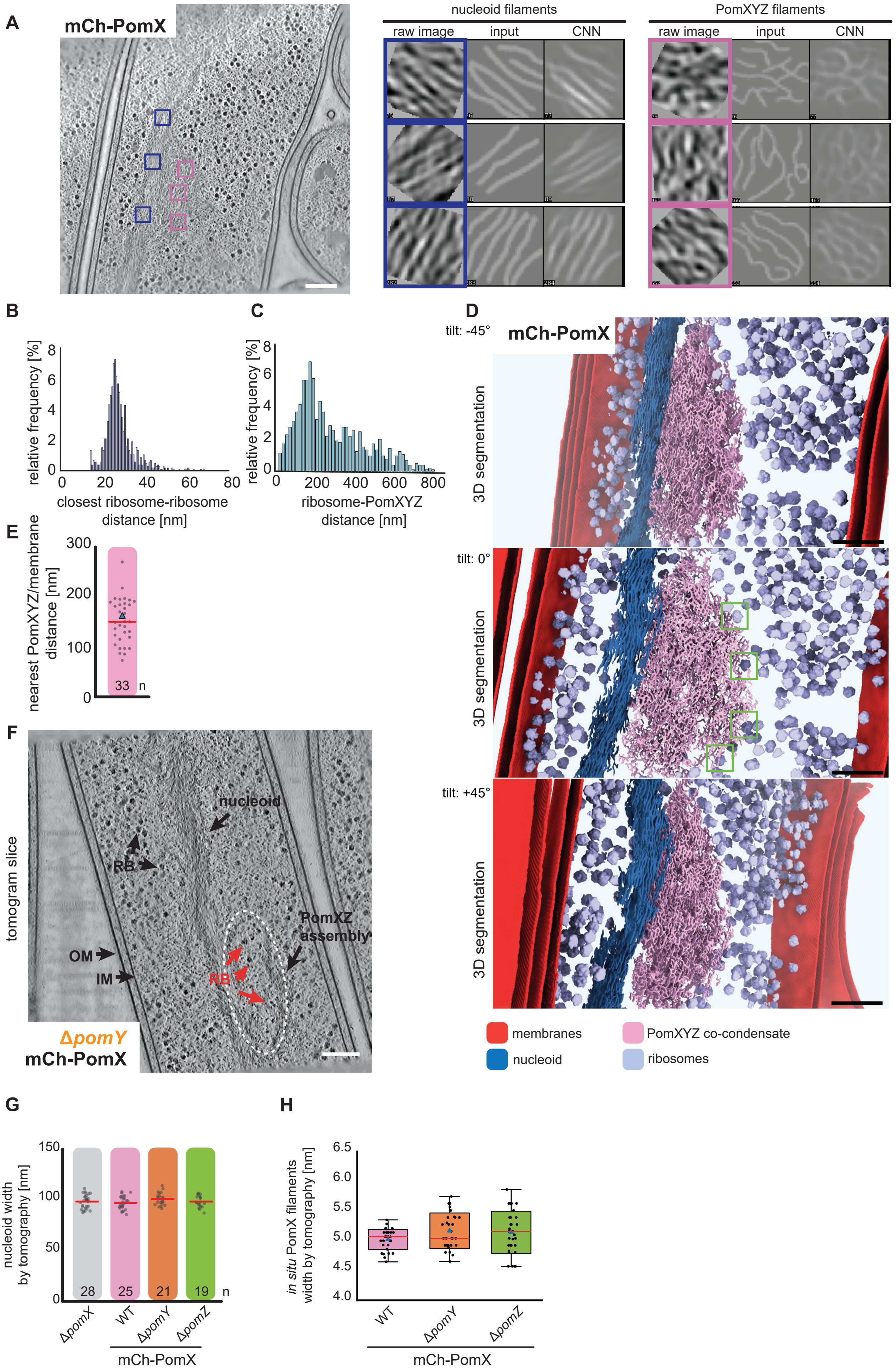

A
mCh-PomX
nucleoid filaments
PomXYZ filaments
raw image
input
CNN
B
relative frequency [%]
closest ribosome-ribosome distance [nm]
C
ribosome-PomXYZ distance [nm]
D
tilt: -45°
tilt: 0°
tilt: +45°
3D segmentation
E
nearest PomXYZ/membrane distance [nm]
33
n
F
tomogram slice
nucleoid
RB
PomXZ assembly
OM
IM
ΔpomY
mCh-PomX
membranes
nucleoid
PomXYZ co-condensate
ribosomes
G
nucleoid width by tomography [nm]
28
25
21
19
ΔpomX
WT
ΔpomY
ΔpomZ
mCh-PomX
H
in situ PomX filaments width by tomography [nm]
WT
ΔpomY
ΔpomZ
mCh-PomX

Figure S2. Analysis of Pom assemblies in the *in situ* cellular environment.
(A) Examples of EMAN2 training boxes of filaments. Blue boxes indicate nucleoid areas for training. Magenta boxes indicate PomXYZ assembly areas for training. From left to right, the columns are the boxed subtomograms, the manual segmentation provided for training, and the CNN segmentation output results.
(B) Ribosome centre-to-centre nearest distance distribution.
(C) Distribution of distance between ribosome and PomXYZ co-condensate.
(D) Different Y-axis orientation views of zoomed-in 3D segmentation from the same image as shown in Fig. 2A at indicated tilt angles. Green boxes indicate ribosomes in close proximity to the PomXYZ co-condensate. Scale bars 100nm.
(E) Distribution of distances from PomXYZ co-condensates to the nearest membrane. 33 tomograms were analysed. Teal triangle indicates the mean, red line is the median.
(F) Example of tomogram with PomXZ assembly showing ribosomes (red arrows) penetrating into the assembly. White dashed circle indicates boundary of the PomXZ assembly. Scale bar 100nm.
(G) Nucleoid width measured from tomograms from cells of indicated genotypes. Red lines indicate the median.
(H) *In situ* PomX filament width measured from tomograms. Whiskers indicate maximum and minimum, box indicate standard deviation, red line indicate median, dark blue triangle indicate mean.

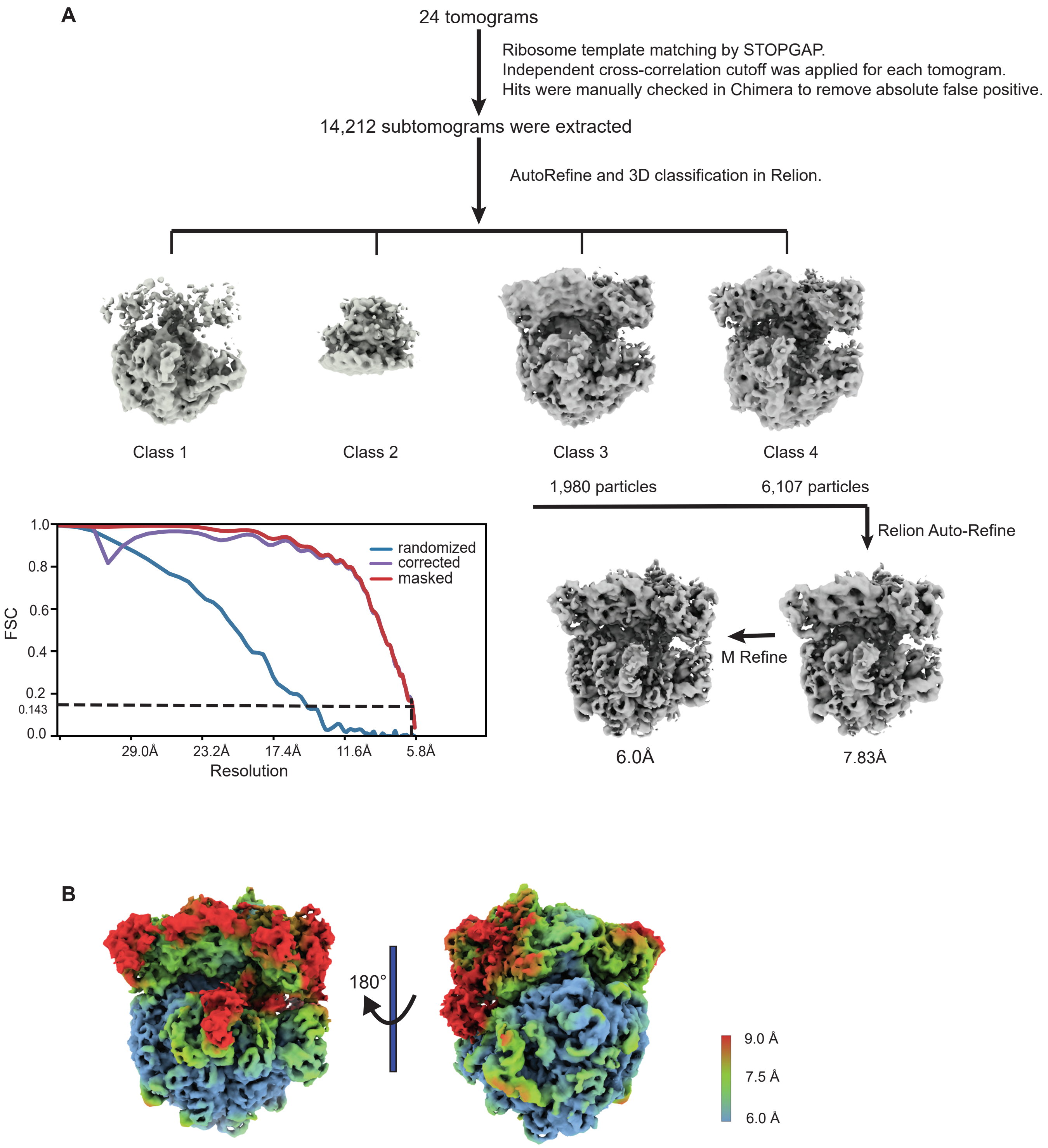
A
24 tomograms
Ribosome template matching by STOPGAP.
Independent cross-correlation cutoff was applied for each tomogram.
Hits were manually checked in Chimera to remove absolute false positive.
14,212 subtomograms were extracted
AutoRefine and 3D classification in Relion.
Class 1
Class 2
Class 3
Class 4
1,980 particles
6,107 particles
Relion Auto-Refine
M Refine
6.0Å
7.83Å
FSC
Resolution
randomized
corrected
masked
1.0
0.8
0.6
0.4
0.2
0.143
0.0
29.0Å
23.2Å
17.4Å
11.6Å
5.8Å
B
180°
9.0 Å
7.5 Å
6.0 Å

Figure S3. Workflow for the analysis of the *M. xanthus* ribosome from cryo-ET dataset.
(A) 24 high-quality tomograms were used to analyse the *in situ* structure of ribosomes. Ribosome template matching was performed in STOPGAP. Each tomogram's template matching result was verified in Chimera and cross-correlation cutoff was set individually. Ultimately, 14,212 ribosome sub-tomograms were extracted. Using Relion's Auto-Refine and 3D classification, classes 3 and 4 were selected with 8,087 particles, representing 56.9% of the total particles number. After Relion Auto-Refine, the data was imported into M for geometric and CTF refinement, achieving an *in situ* ribosome resolution of 6Å (FSC=0.143).
(B) A 6Å *in situ M. xanthus* ribosome map with local resolution, as indicated on the right.

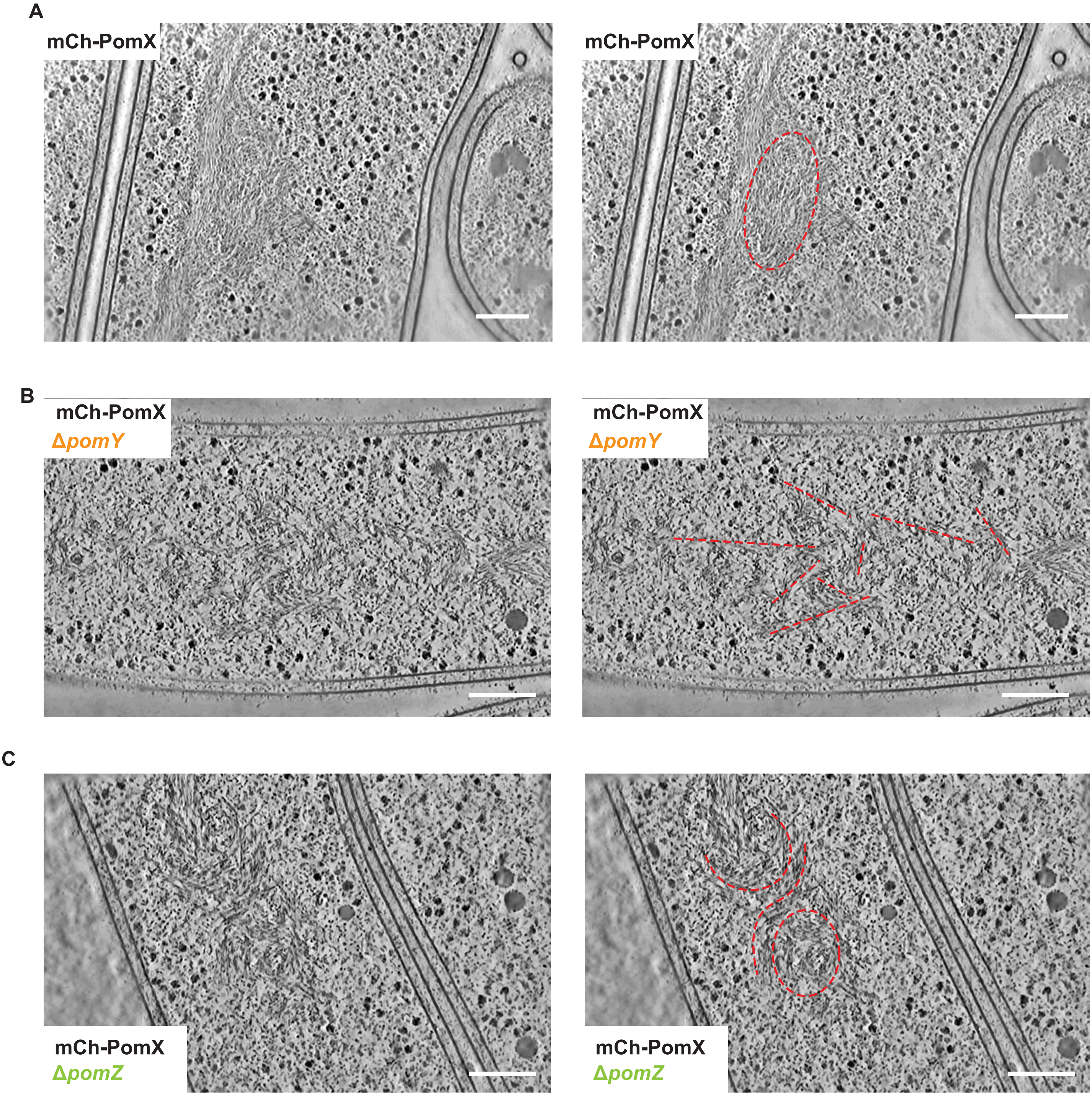
A
mCh-PomX
mCh-PomX
B
mCh-PomX
ΔpomY
mCh-PomX
ΔpomY
C
mCh-PomX
ΔpomZ
mCh-PomX
ΔpomZ

Figure S4. PomX filaments in different Pom assemblies *in situ*.

(A) Left: Zoomed-in view of PomXYZ co-condensate. Right: Same image as left, red dashed circle indicates area are in which the PomX filaments adopt a vortex-like pattern. Scale bar 500nm.

(B) Left: Zoomed-in view of of PomXZ assembly. Right: Same image as left, red dashed lines indicate PomX filaments with a relatively straight orientation. Scale bar 500nm.

(C) Left: Zoomed-in view of PomXY assembly. Right: Same image as left, red dashed lines indicate areas in which the PomX filaments adopt a vortex-like pattern. Scale bar 500nm.

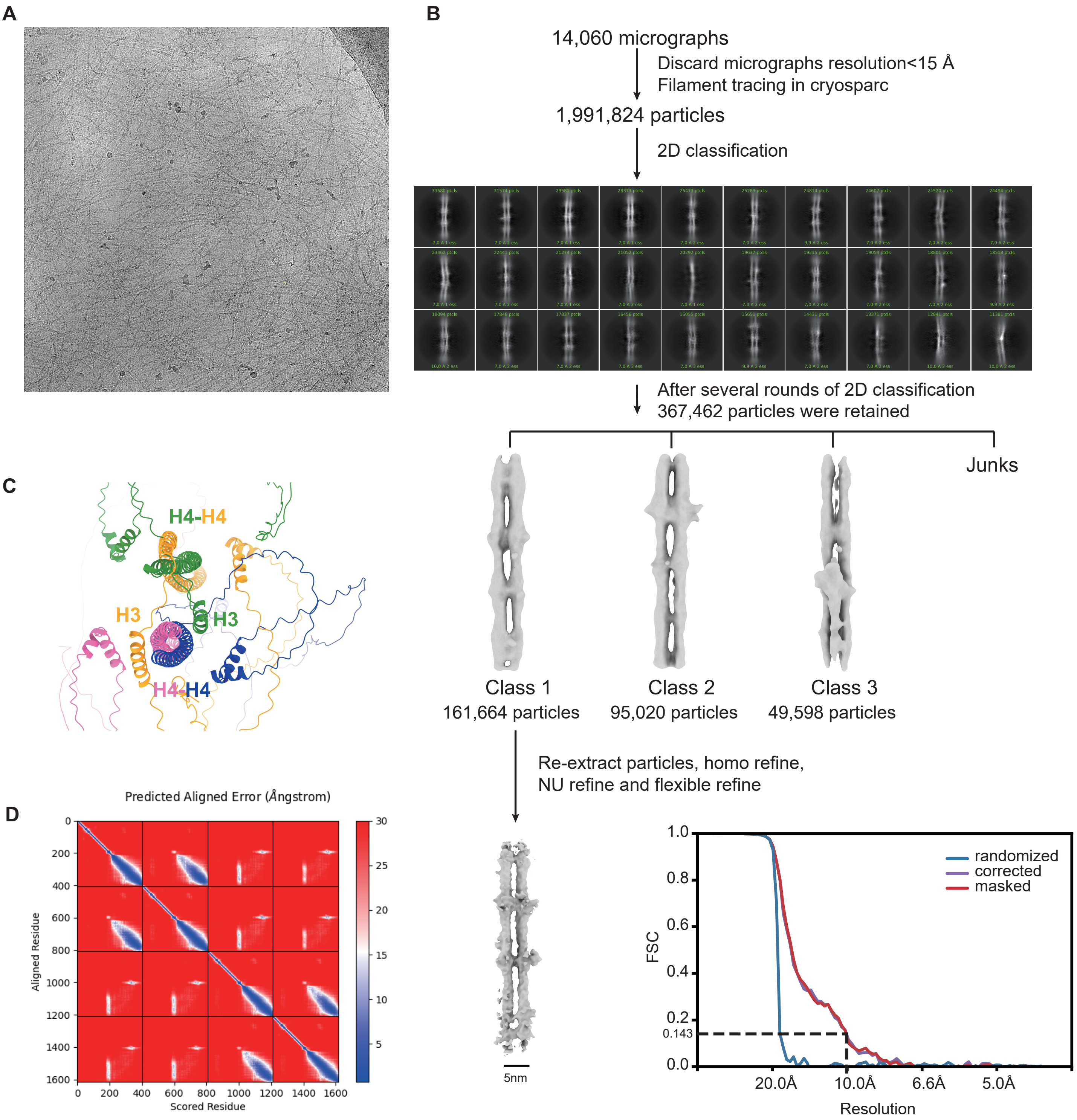
A
B
14,060 micrographs
Discard micrographs resolution<15 Å
Filament tracing in cryosparc
1,991,824 particles
2D classification
After several rounds of 2D classification
367,462 particles were retained
Junks
Class 1
161,664 particles
Class 2
95,020 particles
Class 3
49,598 particles
Re-extract particles, homo refine,
NU refine and flexible refine
5nm
FSC
randomized
corrected
masked
0.143
20.0Å
10.0Å
6.6Å
5.0Å
Resolution
C
H4-H4
H3
H3
H4-H4
D
Predicted Aligned Error (Ångstrom)
Aligned Residue
Scored Residue

Figure S5. Workflow for cryo-EM single particle analysis of the PomX filament.
(A) An example of Cryo-EM micrograph of purified PomX-His_6.
(B) A total number of 1,991,824 particles were picked and extracted from 14,060 micrographs. Following 2D classification, the formation of parallel bundles was distinctly observed. After several rounds of 2D classification, 367,462 particles were retained for further 3D classification. Class 1, comprising of 44% with a total of 161,664 particles was selected, underwent subsequent homogeneous refinement, non-uniform refinement, and flexible refinement. The map underwent post-processing and polishing, yielding a final map with an overall resolution of 10Å (FSC=0.143).
(C) Side view of the predicted model of PomX tetramer used in Fig. 4C, the H3 helices in one dimer interact directly with the opposing H4-H4 coiled-coil structure. The N-terminal 185 residues were modelled with low confidence and are likely not interacting with the H4-H4 rigid body.
(D) Predicted Aligned Error (PAE) representation of the predicted tetrameric PomX configuration used in Fig. 4B-C. The positional error for every residue pair is indicated by colour (0Å in dark blue, 30Å in red). The H4-H4 coiled-coil assemblies are predicted with low error (Domain boundaries: H4: 216-404, 620-808, 1024-1212, 1428-1616. H3: 189-204, 593-608, 997-1012, 1401-1416), while the N-terminal 185 residues for each molecule are predicted with high positional variance.

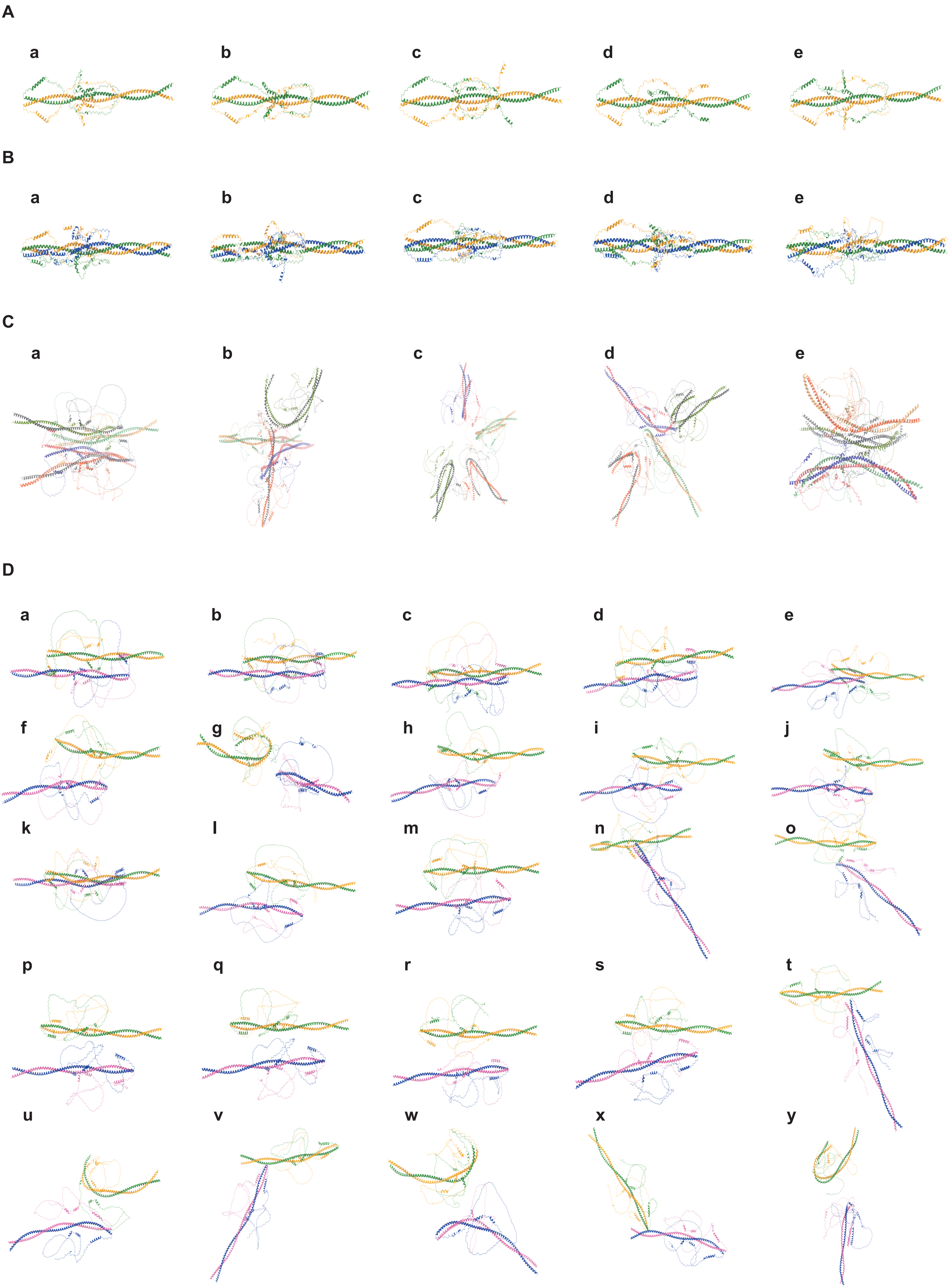
A
a
b
c
d
e
B
a
b
c
d
e
C
a
b
c
d
e
D
a
b
c
d
e
f
g
h
i
j
k
l
m
n
o
p
q
r
s
t
u
v
w
x
y

Figure S6. AlphaFold-Multimer predicted structural models of different PomX oligomeric states
(A) a-e. AlphaFold-Multimer predicted structural models of PomX homodimer. Proposed structures are coloured by chains.
(B) a-e. AlphaFold-Multimer predicted structural models of PomX homotrimer. Proposed structures are coloured by chains.

(C) a-e. AlphaFold-Multimer predicted structural models of PomX homooctamer. Proposed structures are coloured by chains.
(D) a-y. AlphaFold-Multimer predicted structural models of PomX homotetramer, coloured by chains. Only the rigid bodies of models a and b could be fitted into the cryo-EM SPA density map. Other models could not be fitted into the cryo-EM SPA density map accurately. The main differences between models a and b lie in the unstructured N-terminus regions. In this study, model a was selected as the structural model fitted in cryo-EM SPA density map, as shown in Fig. 4B-C.

Table S1. Cryo-ET data collection, refinement statistics for *in situ* 70S ribosome

	70S ribosome (EMD-50504)
**Data collection and processing**	
Magnification	42000
Voltage (kV)	300
Electron exposure (e–/Å^2)	130 to 140
Defocus range (μm)	-4.5 to -5
Pixel size (Å)	2.93
Symmetry imposed	C1
Initial particle images (no.)	14,212
Final particle images (no.)	8,087
Map resolution (Å)<br>FSC threshold 0.143	6.0
Map resolution range (Å)	6 to 9
Map sharpening *B* factor (Å^2)	-77
**Refinement**	
Initial model used (PDB code)	
Model resolution (Å)<br>FSC threshold	
Model resolution range (Å)	
Model composition<br>Non-hydrogen atoms<br>Protein residues<br>Ligands	
B factors (Å^2)<br>Protein<br>Ligand	
R.m.s. deviations<br>Bond lengths (Å)<br>Bond angles (°)	
Validation<br>MolProbity score<br>Clashscore<br>Poor rotamers (%)	
Ramachandran plot<br>Favored (%)<br>Allowed (%)<br>Disallowed (%)	

Table S2: Cryo-EM data collection, refinement statistics for PomX filament

	PomX filament (EMD-50515)
**Data collection and processing**	
Magnification	130000
Voltage (kV)	300
Electron exposure (e–/Å^2)	40 to 60
Defocus range (μm)	-1 to -3.5
Pixel size (Å)	1.09
Symmetry imposed	C1
Initial particle images (no.)	1,991,224
Final particle images (no.)	161,664
Map resolution (Å)<br>FSC threshold 0.143	10.0
Map resolution range (Å)	10 to 35
Map sharpening *B* factor (Å^2)	-100
**Refinement**	
Initial model used (PDB code)	
Model resolution (Å)<br>FSC threshold	
Model resolution range (Å)	
Model composition<br>Non-hydrogen atoms<br>Protein residues<br>Ligands	
B factors (Å^2)<br>Protein<br>Ligand	
R.m.s. deviations<br>Bond lengths (Å)<br>Bond angles (°)	
Validation<br>MolProbity score<br>Clashscore<br>Poor rotamers (%)	
Ramachandran plot<br>Favored (%)<br>Allowed (%)<br>Disallowed (%)	

Legends to Supplementary Movies 1 to 3

Movie S1.
Sequential slices back and forth through a representative tomogram in cross-section view and the 3D rendering models for a cell expressing mCh-PomX.

Movie S2.
Sequential slices back and forth through a representative tomogram in cross-section view and the 3D rendering models for a Δ*pomY* cell expressing mCh-PomX.

Movie S3.
Sequential slices back and forth through a representative tomogram in cross-section view and the 3D rendering models for a Δ*pomZ* cell expressing mCh-PomX.

Supplementary References

1. B. Ramm *et al.*, Biomolecular condensate drives polymerization and bundling of the bacterial tubulin FtsZ to regulate cell division. *Nat. Commun.* **14**, 3825 (2023).

2. A. Paintdakhi *et al.*, Oufti: an integrated software package for high-accuracy, high-throughput quantitative microscopy analysis. *Mol. Microbiol.* **99**, 767-777 (2016).

3. D. Schumacher, A. Harms, S. Bergeler, E. Frey, L. Søgaard-Andersen, PomX, a ParA/MinD ATPase activating protein, is a triple regulator of cell division in Myxococcus xanthus. *eLife* **10**, e66160 (2021).

4. S. Q. Zheng *et al.*, MotionCor2: anisotropic correction of beam-induced motion for improved cryo-electron microscopy. *Nat. Methods* **14**, 331-332 (2017).

5. T. O. Buchholz, M. Jordan, G. Pigino, F. Jug, Cryo-CARE: Content-aware image restoration for cryo-transmission electron microscopy data. *I S Biomed Imaging* 10.1109/isbi.2019.8759519, 502-506 (2019).

6. Y. T. Liu *et al.*, Isotropic reconstruction for electron tomography with deep learning. *Nat. Commun.* **13**, 6482 (2022).

7. S. W. Zheng *et al.*, AreTomo: An integrated software package for automated marker-free, motion-corrected cryo-electron tomographic alignment and reconstruction. *J Struct Biol-X* **6**, 100068 (2022).

8. J. R. Kremer, D. N. Mastronarde, J. R. McIntosh, Computer visualization of three-dimensional image data using IMOD. *J Struct Biol-X* **116**, 71-76 (1996).

9. D. N. Mastronarde, S. R. Held, Automated tilt series alignment and tomographic reconstruction in IMOD. *J. Struct Biol.* **197**, 102-113 (2017).

10. M. Chen *et al.*, Convolutional neural networks for automated annotation of cellular cryo-electron tomograms. *Nat. Methods* **14**, 983-985 (2017).

11. W. Wan, S. Khavnekar, J. Wagner, STOPGAP, an open-source package for template matching, subtomogram alignment, and classification. *bioRxiv* 10.1101/2023.12.20.572665, 10.1101/2023.1112.1120.572665 (2023).

12. N. R. James, A. Brown, Y. Gordiyenko, V. Ramakrishnan, Translational termination without a stop codon. *Science* **354**, 1437-1440 (2016).

13. E. F. Pettersen *et al.*, UCSF Chimera--a visualization system for exploratory research and analysis. *J Comput Chem* **25**, 1605-1612 (2004).

14. T. Hrabe *et al.*, PyTom: A python-based toolbox for localization of macromolecules in cryo-electron tomograms and subtomogram analysis. *J Struct Biol* **178**, 177-188 (2012).

15. D. Tegunov, P. Cramer, Real-time cryo-electron microscopy data preprocessing with Warp. *Nat Methods* **16**, 1146-1152 (2019).

16. D. Kimanius, L. Y. Dong, G. Sharov, T. Nakane, S. H. W. Scheres, New tools for automated cryo-EM single-particle analysis in RELION-4.0. *Biochem J* **478**, 4169-4185 (2021).

17. J. Zivanov *et al.*, New tools for automated high-resolution cryo-EM structure determination in RELION-3. *Elife* **7**, e42166 (2018).

18. A. Martinez-Sanchez, I. Garcia, S. Asano, V. Lucic, J. J. Fernandez, Robust membrane detection based on tensor voting for electron tomography. *J Struct Biol-X* **186**, 49-61 (2014).

19. E. F. Pettersen *et al.*, UCSF ChimeraX: Structure visualization for researchers, educators, and developers. *Protein Sci* **30**, 70-82 (2021).

20. D. Tegunov, L. Xue, C. Dienemann, P. Cramer, J. Mahamid, Multi-particle cryo-EM refinement with M visualizes ribosome-antibiotic complex at 3.5 Å in cells. *Nat Methods* **18**, 186-193 (2021).

21. M. Schemper, Generalized Kruskal-Wallis tests for comparing K-Samples subject to censoring. *S. Afr. Stat. J.* **17**, 1-11 (1983).

22. D. Schumacher *et al.*, The PomXYZ proteins self-organize on the bacterial nucleoid to stimulate cell division. *Dev. Cell* **41**, 299-314 e213 (2017).

23. A. Punjani, J. L. Rubinstein, D. J. Fleet, M. A. Brubaker, cryoSPARC: algorithms for rapid unsupervised cryo-EM structure determination. *Nat. Methods* **14**, 290–296 (2017).

24. A. Punjani, D. J. Fleet, 3DFlex: determining structure and motion of flexible proteins from cryo-EM. *Nat. Methods* **20**, 860-+ (2023).

25. J. Jumper *et al.*, Highly accurate protein structure prediction with AlphaFold. *Nature* **596**, 583-589 (2021).

26. R. Evans *et al.*, Protein complex prediction with AlphaFold-Multimer. *bioRxiv* **2021.10.04.463034** (2022).
